# Predictive factors and outcomes of immune-related adverse events in Chinese patients treated with immune checkpoint inhibitors: a real-world retrospective study

**DOI:** 10.3389/fimmu.2025.1655724

**Published:** 2025-09-23

**Authors:** Haodong Kong, Rongjia Xie, Zhuqing Liu

**Affiliations:** ^1^ Department of Oncology, Shanghai Tenth People’s Hospital, Tongji University School of Medicine, Shanghai, China; ^2^ Tongji University Cancer Center, Shanghai, China; ^3^ Department of Orthopedics, Shanghai General Hospital, Shanghai Jiao Tong University School of Medicine, Shanghai, China; ^4^ Shanghai Cancer Comprehensive Center, Shanghai General Hospital, Shanghai Jiao Tong University School of Medicine, Shanghai, China

**Keywords:** immune checkpoint inhibitors (ICIs), immune-related adverse events (irAEs), predictive factors, efficacy, Chinese agents

## Abstract

**Background:**

The widespread clinical application of immune checkpoint inhibitors (ICIs) has brought immune-related adverse events (irAEs) to the forefront of oncology management. However, systematic investigations focusing on the efficacy and irAE profiles of ICIs locally developed in China (Chinese ICIs) in real-world settings remain limited. This study aimed to explore predictive factors and outcomes of irAEs in patients predominantly treated with Chinese ICIs.

**Methods:**

In this single-center retrospective study, 206 patients with solid tumors treated with ICIs between 2020 and 2024 were included, with 87.9% receiving Chinese ICIs. Multivariate regression analyses were conducted to identify predictors of irAEs and factors associated with progression-free survival (PFS). Clinical characteristics, inflammatory biomarkers, cytokine levels, and cardiac function parameters were comprehensively evaluated.

**Results:**

Younger age (p=0.04) and the brain metastases (p=0.03) were associated with a higher incidence of irAEs. Organ-specific irAEs showed distinct predictors: hepatitis with younger age, hepatitis B virus (HBV) infection, hepatic malignancy, multisystem irAEs, and elevated IL-10; myocarditis with multisystem irAEs, systemic inflammation markers (white blood cell count, WBC; neutrophil-to-lymphocyte ratio, NLR; systemic immune-inflammation index, SII; systemic inflammation response index, SIRI), and cardiac function indicators (left ventricular ejection fraction, LVEF; QRS duration); thyroiditis with multisystem irAEs and reduced IL-6; while pneumonitis with elevated platelet count (PLT) and IL-6. The type of irAEs was not associated with PFS.

**Conclusions:**

Younger age and brain metastases were associated with a higher incidence of irAEs. Organ-specific irAEs were characterized by distinct sets of clinical and laboratory predictors. Inflammatory markers correlated with poorer PFS. The type of irAEs was not associated with PFS. Chinese ICIs were not independently associated with an increased risk of specific organ toxicities.

## Introduction

In recent years, cancer immunotherapy has achieved significant breakthroughs. Immune checkpoint inhibitors (ICIs), particularly antibodies targeting PD-1/PD-L1 and CTLA-4, have demonstrated remarkable efficacy in advanced malignancies such as gastric cancer, colorectal cancer, and non-small cell lung cancer (NSCLC) (1–3). With the growing body of evidence supporting the combination of immunotherapy and chemotherapy, ICIs are increasingly being utilized not only as later-line treatments but also in first-line and combination regimens (4, 5). Moreover, several ICIs locally developed in China (Chinese ICIs) have also entered the international market. For reference, tislelizumab has been approved by the European Medicines Agency (EMA) and the United States Food and Drug Administration (FDA), mainly for the treatment of esophageal cancer (6). Toripalimab has received FDA approval for use in combination with gemcitabine and cisplatin as first-line therapy for patients with advanced recurrent or metastatic nasopharyngeal carcinoma, and it is now available in the United States and the European Union (7). Sintilimab and camrelizumab are not yet approved outside of China, but emerging studies have demonstrated their potential value in clinical practice (8, 9).

As ICIs become widely adopted in cancer therapy, the management of immune-related adverse events (irAEs) has emerged as a critical component of oncologic care. IrAEs encompass more than 70 pathological manifestations, with a spectrum ranging from mild discomfort to life-threatening conditions, potentially affecting virtually any organ system (10). Although the underlying pathogenesis of irAEs remains incompletely understood, T-cell hyperactivation is widely recognized as a central mechanism. By blocking CTLA-4 and PD-1/PD-L1 pathways, ICIs release immune suppression and enhance T-cell activation, promoting cytotoxic responses and proinflammatory cytokine secretion. However, this mechanism alone cannot fully account for the organ-specific manifestations of irAEs. To explain these organ-specific patterns, emerging studies have proposed several additional mechanisms. One mechanism suggests that the reactivation of tissue-resident memory T cells may play a role in the development of ICI-associated colitis (11). Additionally, microbiota-dependent activation of CD4^+^ T cells through Fc-γ receptor engagement has been implicated in CTLA-4 inhibitor-related colitis (12). Another study suggests that ICI-induced myocarditis may be driven by IFN-γ-mediated expansion of inflammatory macrophages (13). Furthermore, the activation of B cells and plasma cells could lead to the production of autoantibodies, such as anti-CD74, which, in conjunction with activated T cells, may contribute to the development of ICI-associated pneumonitis (14). These findings offer partial insights into the organ-specific mechanisms of irAEs, though further elucidation is warranted.

Common irAEs include dermatologic inflammation, gastrointestinal toxicity, and endocrine dysfunction. Although rarer, pneumonitis, myocarditis, and neurotoxicity warrant special clinical attention due to their potential severity (15). Generally, the incidence of irAEs is lower with ICI monotherapy, but the risk is influenced by ICI class, treatment regimens, and tumor type (16). Studies suggest that compared to PD-1/PD-L1 inhibitors, CTLA-4 inhibitors and combination therapies are associated with significantly higher irAE rates and severity (17). While mild irAEs may not necessitate treatment interruption, they require careful monitoring; moderate-to-severe irAEs can result in significant organ dysfunction or even mortality, underscoring the importance of early recognition and intervention to ensure treatment safety.

Currently, Chinese ICIs, which are immune checkpoint inhibitors locally developed in China, have increasingly become first-line options for patients with advanced malignancies (8, 18–20). Several prospective clinical trials have also confirmed the significant efficacy and promising application of Chinese ICIs in adjuvant and neoadjuvant settings (21–23). However, comprehensive real-world studies evaluating the efficacy and irAE profiles of Chinese ICIs remain scarce. In particular, there is a lack of in-depth analyses regarding predictors of irAEs among patients primarily treated with Chinese ICIs. Therefore, this retrospective study was conducted to assess the occurrence of irAEs and to identify potential predictive factors among patients receiving predominantly Chinese ICIs, with the aim of informing clinical application and enhancing the safety management of Chinese ICIs.

## Methods

### Study design and patient population

This study is a retrospective analysis aimed at identifying the risk factors and potential predictors of irAEs in cancer patients treated with ICIs. Patients treated with ICIs at our institution between January 1, 2020, and December 31, 2024, were included based on the following inclusion and exclusion criteria. The inclusion criteria were: age ≥ 18 years, regardless of gender; completion of at least one cycle of ICI treatment; pathologically confirmed advanced solid tumor; availability of complete baseline clinical data (such as age, gender, medical history, treatment regimen, irAEs occurrence); follow-up duration ≥ 3 months. Exclusion criteria included: a history of severe autoimmune diseases (such as systemic lupus erythematosus, myasthenia gravis) or severe organ dysfunction; use of immunosuppressants or corticosteroids within 3 months prior to ICI treatment; lack of critical clinical data such as missing baseline data or insufficient follow-up time (<3 months). Baseline characteristics (including age, gender, tumor type, medical history, treatment regimen, irAEs occurrence) and laboratory data were collected from the electronic medical records system.

### Data collection

Clinical and pathological data from patients who received ICI treatment were retrieved by reviewing the electronic medical records. Baseline characteristics included age, gender, body mass index (BMI), medical history (smoking status, hypertension, diabetes, coronary heart disease, exposure to antihypertensive or antidiabetic drugs), tumor type, metastatic sites, ICI regimen, pre-existing treatment (previous treatments were classified as chemotherapy only, radiotherapy only, both chemotherapy and radiotherapy, or none, referring to no prior chemotherapy or radiotherapy, regardless of surgery history), and concomitant medications. Laboratory data collected included complete blood counts (white blood cell count (WBC), absolute neutrophil count (ANC), absolute eosinophil count (AEC), absolute monocyte count (AMC), absolute lymphocyte count (ALC), platelet count (PLT), neutrophil-to-lymphocyte ratio (NLR), lymphocyte-to-monocyte ratio (LMR), platelet-to-lymphocyte ratio (PLR), systemic immune-inflammation index (SII), systemic inflammatory response index (SIRI), serum lactate dehydrogenase (LDH), C-reactive protein (CRP), and cytokines (IL-1, IL-2, IL-6, IL-10, IL-17). Additionally, electrocardiographic data (including heart rate, HR; PR interval; QRS duration; QRS axis; QT interval; corrected QT interval, QTc; RV5 + SV1 voltage; and RV5 + SV1 amplitude), and echocardiographic data (including left ventricular ejection fraction, LVEF; aortic diameter, AOD; left atrial diameter, LAD; left ventricular end-diastolic diameter, LVDD; left ventricular end-systolic diameter, LVDS; interventricular septum thickness, IVS; left ventricular posterior wall thickness, LVPW; and S-wave peak velocity) were recorded. The above-mentioned laboratory tests and cardiac evaluations were routinely performed before and after ICI initiation to establish baselines and monitor treatment-related changes. Specifically, pre-treatment time points were defined as within one month before starting ICI therapy. Post-treatment time points were selected differently for patients with and without irAEs: for those with irAEs, data were taken from the last assessment prior to irAE diagnosis; for those without irAEs, data were taken at three months after ICI initiation. All laboratory and cardiac evaluation indices in this study are presented as changes from baseline, calculated by subtracting pre-treatment values from post-treatment values and denoted with the delta symbol (Δ), for example, neutrophil-to-lymphocyte ratio (NLR) is reported as ΔNLR.

Treatment response was categorized according to the revised RECIST (version 1.1) criteria as complete response (CR), partial response (PR), stable disease (SD), or progressive disease (PD). Progression-free survival (PFS) was defined as the time from the initiation of ICI treatment until PD or death from any cause. Objective response rate (ORR) was defined as the sum of CR and PR, and disease control rate (DCR) was defined as the sum of CR, PR, and SD. Diagnosis of irAEs was based on the Common Terminology Criteria for Adverse Events (CTCAE, version 5.0) and was systematically evaluated by oncologists at our institution.

### Statistical analysis

Data analysis was performed using R version 4.4.2 and GraphPad Prism 8.0. For baseline characteristic analysis, continuous variables were presented as median (IQR), and group comparisons were performed using the t-test (for normally distributed data) or the Wilcoxon rank-sum test (for non-normally distributed data). The normality of data distribution was assessed using the Shapiro–Wilk test. Categorical variables were expressed as the number of patients (n) and percentage (%) and were compared using the Chi-square test. Missing laboratory values were handled by complete-case analysis, excluding patients with missing data for that variable. Univariate and multivariate Cox regression analyses were performed with potential relevant continuous or categorical variables as independent variables, PFS as the time variable, and PD as the outcome to evaluate influencing factors. Univariate and multivariate logistic regression analyses were conducted with potential relevant continuous or categorical variables as independent variables and irAEs as the dependent variable to explore their influencing factors. For Cox and logistic regression analyses, variables with p < 0.1 in univariate analysis were included in the initial multivariate model. The variables assessed covered four main domains: (1) clinical characteristics; (2) inflammatory biomarkers; (3) cytokine levels; and (4) cardiac function parameters. Survival analysis was conducted using the Kaplan-Meier method to generate survival curves and assess survival differences. The final study figures were created using GraphPad Prism, and statistical significance was evaluated (a two-sided p-value ≤ 0.05 was considered statistically significant).

## Results

### Patient characteristics

**Figure 1:**
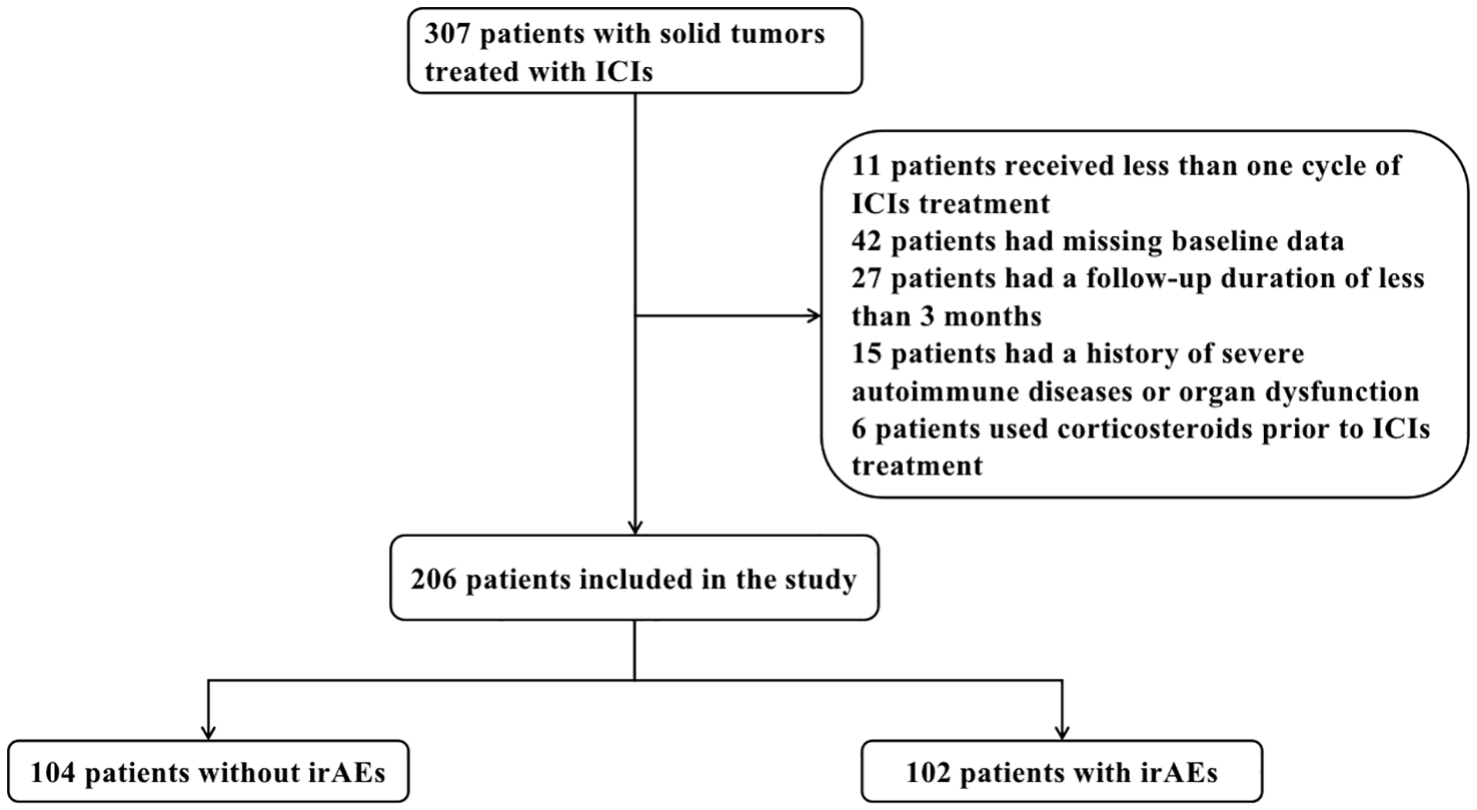
Flowchart of the study design. ICIs, immune checkpoint inhibitors; irAEs, immune-related adverse events.

As the flowchart presented in **Figure 1**, this study included a total of 206 patients with solid tumors who were treated with ICIs. The baseline characteristics of the patients are summarized in **Table 1**. Overall, the majority of these patients received Chinese ICIs (181 patients, 87.9%), with the top four drugs being sintilimab (77 patients, 37.3%), camrelizumab (42 patients, 20.3%), tislelizumab (25 patients, 12.1%), and toripalimab (19 patients, 9.2%). Among the 206 patients, 104 patients (50.5%) did not experience irAEs, while 102 patients (49.5%) developed irAEs. The most common organ-specific irAEs among those who developed irAEs were hepatitis (36 cases, 35.3%), myocarditis (22 cases, 21.6%), thyroiditis (14 cases, 13.7%), pneumonitis (11 cases, 10.8%), and hypophysitis (8 cases, 7.8%). Additionally, 12 patients (11.8%) experienced two or more irAEs concurrently.

**Table 1:** Baseline characteristics of patients with or without irAEs.

Characteristics	Patient group		*P* value
	Without irAEs (n=104)	With irAEs (n=102)	
Age at start of ICIs, y, median (IQR)	68.5 (62.0-73.0)	66.0 (59.0-72.0)	0.04
BMI, kg/m^2^, median (IQR)	21.8 (19.7-23.6)	22.1 (19.9-24.0)	0.56
Sex, n (%)			
Male	69 (66.3)	64 (62.7)	0.69
Female	35 (33.7)	38 (37.3)	
BMI, kg/m², median (IQR)	21.8 (19.7-23.6)	22.1 (19.9-24.0)	0.56
Medical history, n (%)			
Smoking status	12 (11.5)	11 (10.8)	1.00
Hypertension	35 (33.7)	28 (27.5)	0.42
Diabetes	15 (14.4)	13 (12.7)	0.88
Coronary heart disease	6 (5.8)	6 (5.9)	1.00
Antihypertensive drug exposure	23 (22.1)	18 (17.6)	0.53
Antidiabetic drug exposure	9 (8.7)	5 (4.9)	0.43
Tumor category, n (%)			
Esophageal	14 (13.5)	6 (5.9)	0.11
Gastric	26 (25.0)	16 (15.7)	0.14
Colorectal	18 (17.3)	20 (19.6)	0.81
Hepatic	2 (1.9)	7 (6.9)	0.10
Pancreatic	5 (4.8)	3 (2.9)	0.72
Pulmonary	19 (18.3)	25 (24.5)	0.36
Renal	4 (3.9)	4 (3.9)	1.00
Melanoma	7 (6.7)	8 (7.8)	0.97
Metastatic site, n (%)			
Liver	28 (26.9)	31 (30.4)	0.69
Lung	31 (29.8)	31 (30.4)	1.00
Bone	20 (19.2)	19 (18.6)	1.00
Brain	2 (1.9)	10 (9.8)	0.03
ICI, n (%)			
Sintilimab	40 (38.5)	37 (36.3)	0.86
Camrelizumab	19 (18.3)	23 (22.5)	0.56
Tislelizumab	12 (11.5)	13 (12.7)	0.96
Toripalimab	10 (9.6)	9 (8.8)	1.00
Pembrolizumab	8 (7.7)	10 (9.8)	0.77
Pre-existing treatment, n (%)			
Chemotherapy	63 (60.6)	68 (66.7)	0.45
Radiotherapy	1 (1.0)	0 (0.0)	1.00
Chemotherapy plus radiotherapy	6 (5.8)	15 (14.7)	0.06
None	34 (32.7)	19 (18.6)	0.03
Combination therapy, n (%)			
Chemotherapy	44 (42.3)	32 (31.4)	0.14
Radiotherapy	1 (1.0)	0 (0.0)	1.00
Targeted therapy	43 (41.3)	53 (52.0)	0.17
None	16(15.4)	16 (15.7)	1.00

irAEs, immune-related adverse events; ICIs, immune checkpoint inhibitors; BMI, body mass index; IQR, interquartile range.

When comparing the group of patients who did not experience irAEs to those who developed irAEs, significant differences were observed in age (68.5 vs. 66.0, p = 0.04), tumor brain metastasis (2 vs. 10 patients, p = 0.03), and pre-existing treatments before ICI therapy (34 vs. 19 patients, p = 0.03).

### Factors associated with PFS following ICI treatment

We employed a multivariable Cox regression model, adjusting for factors such as age, gender, BMI, and medical history, to analyze risk factors associated with PFS (**Table 2**). The results revealed that the following factors were significantly associated with PFS: age at ICI treatment (HR: 0.97, 95% CI: 0.95-0.98, p < 0.001), liver metastasis (HR: 1.95, 95% CI: 1.17-3.24, p = 0.01), the absence of combination therapy (HR = 0.51, 95% CI: 0.28-0.95, P = 0.03), and the time from ICI treatment to the diagnosis of irAEs (HR: 0.83, 95% CI: 0.74-0.93, p < 0.001). We also analyzed changes in laboratory markers in peripheral blood before and after ICI treatment and found that ΔNLR (HR: 0.83, 95% CI: 0.74-0.93, p < 0.001), ΔPLR (HR: 1.001, 95% CI: 1.000-1.002, p = 0.03), ΔSII (HR: 1.0001, 95% CI: 1.0000-1.0002, p = 0.01), ΔSIRI (HR: 1.05, 95% CI: 1.01-1.09, p = 0.02), and ΔIL-8 (HR: 1.001, 95% CI: 1.000-1.002, p = 0.02) were significantly associated with PFS.

**Table 2:** Univariate and multivariate analysis of factors associated with progression-free survival.

Factors	Univariate analysis			Multivariate analysis		
	HR	95 % CI	*P* value	HR	95 % CI	*P* value
Age at start of ICIs	0.97	[0.96, 0.98]	<0.001	0.97	[0.95, 0.98]	<0.001
Sex	1.21	[0.83, 1.78]	0.32			
BMI	0.97	[0.93, 1.02]	0.30			
Medical history						
Smoking status	1.27	[0.74, 2.18]	0.39			
Hypertension	0.72	[0.48, 1.08]	0.11			
Diabetes	1.04	[0.64, 1.70]	0.87			
Coronary heart disease	0.53	[0.22, 1.29]	0.16			
Antihypertensive drug exposure	0.76	[0.48, 1.21]	0.25			
Antidiabetic drug exposure	0.81	[0.41, 1.59]	0.54			
Metastatic site						
Liver	1.96	[1.33, 2.89]	0.001	1.95	[1.17, 3.24]	0.01
Lung	1.05	[0.73, 1.53]	0.78			
Bone	1.46	[0.97, 2.19]	0.07			
Brain	1.16	[0.57, 2.38]	0.68			
Pre-existing treatment						
Chemotherapy	1.13	[0.78, 1.63]	0.51			
Radiotherapy	2.49	[0.35, 17.95]	0.37			
Chemotherapy plus radiotherapy	0.64	[0.35, 1.17]	0.15			
None	1.06	[0.71, 1.59]	0.76			
Combination therapy						
Chemotherapy	1.20	[0.83, 1.72]	0.33			
Radiotherapy	1.81	[0.25, 13.02]	0.56			
Targeted therapy	1.18	[0.83, 1.68]	0.35			
None	0.50	[0.29, 0.88]	0.02	0.51	[0.28, 0.95]	0.03
Time from ICIs to diagnosis	0.86	[0.78, 0.94]	0.002	0.83	[0.74, 0.93]	<0.001
Blood routine test						
ΔWBC	1.04	[0.99, 1.09]	0.12			
ΔANC	1.08	[1.02, 1.14]	0.01			
ΔAEC	1.19	[0.54, 2.64]	0.67			
ΔAMC	1.06	[0.63, 1.77]	0.83			
ΔALC	1.00	[0.97, 1.03]	0.87			
ΔPLT	1.00	[0.999, 1.003]	0.16			
ΔNLR	1.05	[1.02, 1.08]	<0.001	1.05	[1.02, 1.09]	0.003
ΔLMR	1.00	[0.99, 1.01	0.93			
ΔPLR	1.00	[1.0003, 1.0020]	0.01	1.00	[1.0001, 1.0020]	0.03
ΔSII	1.00	[1.00004, 1.00021]	0.004	1.00	[1.00003, 1.00025]	0.01
ΔSIRI	1.05	[1.01, 1.08]	0.01	1.05	[1.01, 1.09]	0.02
ΔLDH	1.00	[0.9994, 1.0006]	0.99			
ΔCRP	1.01	[1.001, 1.011]	0.01			
Cytokine						
ΔIL-1	1.03	[0.99, 1.07]	0.19			
ΔIL-2	1.03	[0.98, 1.08]	0.31			
ΔIL-6	1.00	[1.00, 1.01]	0.01			
ΔIL-8	1.00	[1.0001, 1.0015]	0.03	1.00	[1.0002, 1.0017]	0.02
ΔIL-10	1.01	[0.99, 1.03]	0.38			
ΔIL-17	1.01	[1.00, 1.02]	0.05			

PFS, progression-free survival; ICIs, immune checkpoint inhibitors; BMI, body mass index; WBC, white blood cell count; ANC, absolute neutrophil count; AEC, absolute eosinophil count; AMC, absolute monocyte count; ALC, absolute lymphocyte count; PLT, platelet count; NLR, neutrophil-to-lymphocyte ratio; LMR, lymphocyte-to-monocyte ratio; PLR, platelet-to-lymphocyte ratio; SII, systemic immune-inflammation index; SIRI, systemic inflammation response index; LDH, lactate dehydrogenase; CRP, C-reactive protein; HR, hazard ratio; 95% CI, 95% confidence interval.

**Figure 2:**
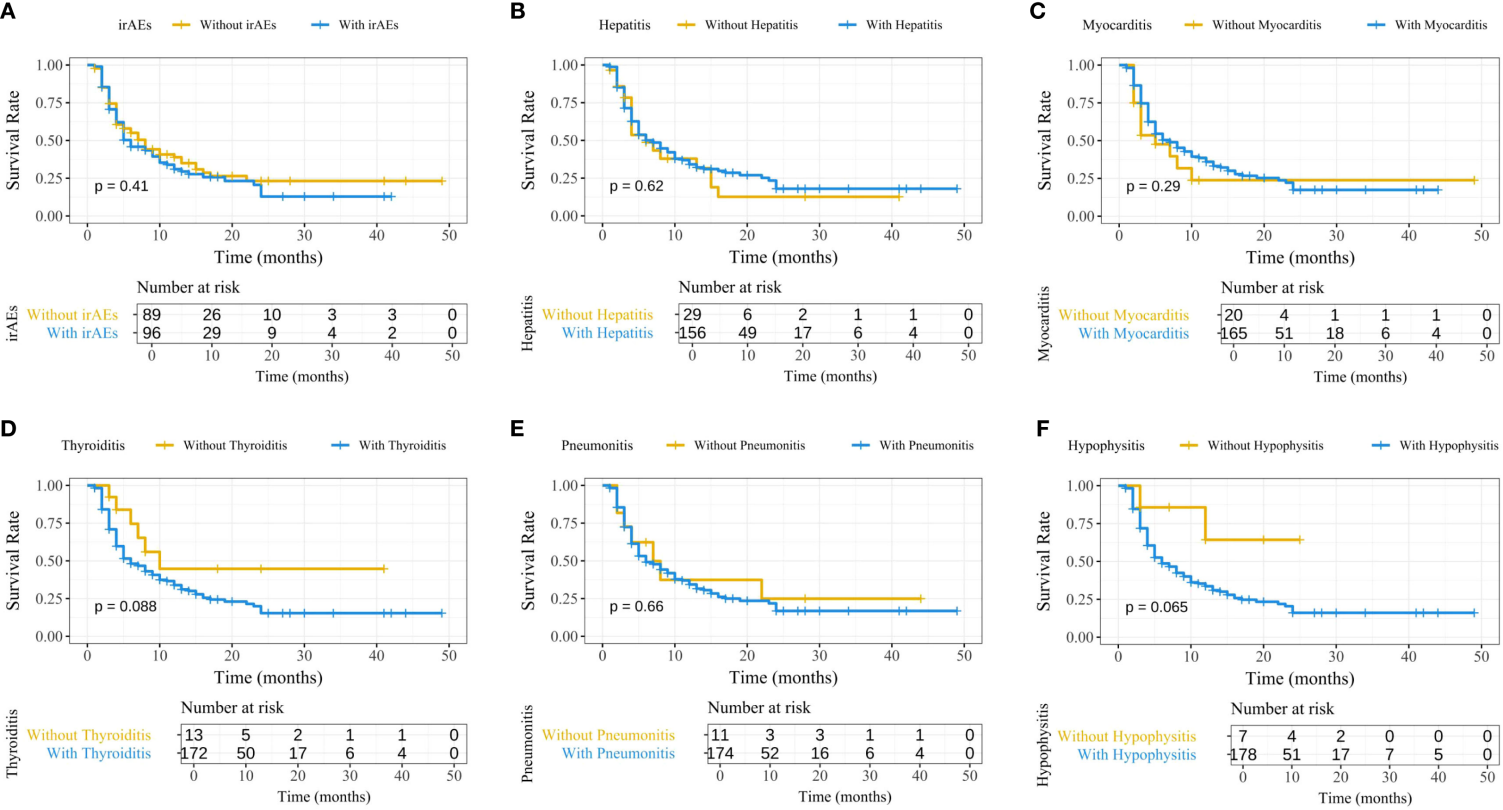
Kaplan-Meier survival curves comparing PFS between patients with and without irAEs. **(A)** Overall irAEs; **(B)** Immune-related hepatitis; **(C)** Immune-related myocarditis; **(D)** Immune-related thyroiditis; **(E)** Immune-related pneumonitis; **(F)** Immune-related hypophysitis. PFS, progression-free survival; irAEs, immune-related adverse events.

To explore the impact of irAEs on PFS, Kaplan-Meier survival curves were generated (**Figure 2**). The results showed no significant differences in PFS between patients with irAEs such as hepatitis, myocarditis, thyroiditis, pneumonitis, and hypophysitis, indicating that none of these irAEs significantly influenced patient survival. To account for immortal time bias, a time-dependent Cox analysis of irAEs and PFS was conducted (**Supplementary Table 1**).

### Predictive factors for irAEs

A multivariable logistic regression model was used to analyze predictive factors for immune-related hepatitis (**Table 3**). Baseline data of the patients revealed that age at ICI treatment (OR: 0.95, 95% CI: 0.92-0.99, p = 0.005), history of prior hepatitis virus infection (OR: 7.59, 95% CI: 2.03-28.40, p = 0.003), primary liver malignancy (OR: 9.78, 95% CI: 1.82-52.49, p = 0.01), and occurrence of two or more irAEs (OR: 6.79, 95% CI: 1.49-30.87, p = 0.01) were significantly associated with the development of immune-related hepatitis. Inflammation-related factors, such as ΔIL-10 (OR: 1.14, 95% CI: 1.05-1.25, p = 0.003), were also significantly associated with hepatitis, while other cytokines, peripheral blood routine markers, LDH, and CRP had no significant impact.

**Table 3:** Univariate and multivariate analysis of factors for patients with immune-related hepatitis.

Factors	Univariate analysis			Multivariate analysis		
	OR	95 % CI	*P* value	OR	95 % CI	*P* value
Age at start of ICIs	0.95	[0.93, 0.98]	0.003	0.95	[0.92, 0.99]	0.005
Sex	0.97	[0.46, 2.01]	0.93			
BMI	1.06	[0.97, 1.16]	0.18			
Medical history						
Smoking status	0.68	[0.15, 2.14]	0.55			
Hypertension	1.00	[0.44, 2.14]	1.00			
Diabetes	0.33	[0.05, 1.16]	0.14			
Coronary heart disease	0.94	[0.14, 3.78]	0.94			
Hepatitis virus infection	5.11	[1.78, 14.50]	0.002	7.59	[2.03, 28.40]	0.003
Liver cancer	6.69	[1.68, 28.39]	0.01	9.78	[1.82, 52.49]	0.01
Metastatic site						
Liver	1.77	[0.82, 3.72]	0.14			
Lung	0.50	[0.19, 1.17]	0.13			
Bone	0.48	[0.14, 1.32]	0.20			
Brain	0.94	[0.14, 3.78]	0.94			
Pre-existing treatment						
Chemotherapy	1.37	[0.65, 3.08]	0.42			
Radiotherapy	——	——	——			
Chemotherapy plus radiotherapy	0.47	[0.07, 1.72]	0.32			
None	0.95	[0.40, 2.12]	0.91			
Combination therapy						
Chemotherapy	0.83	[0.38, 1.74]	0.63			
Radiotherapy	——	——	——			
Targeted therapy	1.35	[0.66, 2.80]	0.41			
None	0.63	[0.18, 1.76]	0.42			
Time from ICIs to diagnosis	1.01	[0.94, 1.10]	0.70			
Co-existing other irAEs	5.47	[1.61, 18.60]	0.01	6.79	[1.49, 30.87]	0.01
ICIs						
Sintilimab	1.08	[0.51, 2.24]	0.84			
Camrelizumab	0.93	[0.35, 2.20]	0.88			
Tislelizumab	0.89	[0.25, 2.53]	0.84			
Toripalimab	1.29	[0.35, 3.84]	0.67			
Best observed response						
Complete response	1.08	[0.06, 7.03]	0.95			
Partial response	0.70	[0.11, 2.66]	0.64			
Stable disease	1.18	[0.53, 2.70]	0.68			
Progressive disease	0.93	[0.38, 2.13]	0.86			
ORR	0.78	[0.18, 2.50]	0.71			
DCR	1.08	[0.47, 2.65]	0.86			
Blood routine test						
ΔWBC	1.03	[0.93, 1.13]	0.59			
ΔANC	1.05	[0.94, 1.17]	0.40			
ΔAEC	1.87	[0.42, 8.74]	0.42			
ΔAMC	0.58	[0.2046, 1.0002]	0.31			
ΔALC	0.80	[0.42, 1.04]	0.50			
ΔPLT	——	——	——			
ΔNLR	1.02	[0.96, 1.07]	0.47			
ΔLMR	——	——	——			
ΔPLR	1.00	[0.999, 1.003]	0.32			
ΔSII	1.00	[0.9999, 1.0003]	0.28			
ΔSIRI	0.98	[0.93, 1.01]	0.19			
ΔLDH	1.00	[1.00, 1.01]	0.03			
ΔCRP	1.00	[0.99, 1.01]	0.36			
Cytokine						
ΔIL-1	1.03	[0.97, 1.12]	0.49			
ΔIL-2	1.04	[0.95, 1.19]	0.48			
ΔIL-6	1.00	[0.99, 1.01]	0.84			
ΔIL-8	1.00	[1.000, 1.002]	0.17			
ΔIL-10	1.12	[1.04, 1.21]	0.005	1.14	[1.05, 1.25]	0.003
ΔIL-17	1.00	[0.98, 1.01]	0.63			

ICIs, immune checkpoint inhibitors; BMI, body mass index; irAEs, immune-related adverse events; CR, complete response; PR, partial response; SD, stable disease; PD, progressive disease; ORR, objective response rate; DCR, disease control rate; WBC, white blood cell count; ANC, absolute neutrophil count; AEC, absolute eosinophil count; AMC, absolute monocyte count; ALC, absolute lymphocyte count; PLT, platelet count; NLR, neutrophil-to-lymphocyte ratio; LMR, lymphocyte-to-monocyte ratio; PLR, platelet-to-lymphocyte ratio; SII, systemic immune-inflammation index; SIRI, systemic inflammation response index; LDH, lactate dehydrogenase; CRP, C-reactive protein; OR, odds ratio; 95% CI, 95% confidence interval.

Logistic regression analysis of risk factors for immune-related myocarditis (**Table 4**) revealed that the occurrence of two or more irAEs (OR: 8.78, 95% CI: 2.03-37.94, p = 0.003) was an independent risk factor for myocarditis. Laboratory markers such as ΔWBC (OR: 1.16, 95% CI: 1.01-1.32, p = 0.04), ΔANC (OR: 1.22, 95% CI: 1.05-1.43, p = 0.01), ΔNLR (OR: 1.11, 95% CI: 1.03-1.21, p = 0.01), ΔSII (OR: 1.0003, 95% CI: 1.0001-1.0006, p = 0.004), and ΔSIRI (OR: 1.10, 95% CI: 1.01-1.21, p = 0.03) were all associated with an increased risk of myocarditis. No predictive role was found for LDH, CRP, or cytokines in myocarditis. We also analyzed electrocardiogram and echocardiogram indices, and found that ΔQRS duration (OR: 1.06, 95% CI: 1.002-1.124, p = 0.04) and ΔLVEF (OR: 0.84, 95% CI: 0.74-0.96, p = 0.01) had predictive value.

**Table 4:** Univariate and multivariate analysis of factors for patients with immune-related myocarditis.

Factors	Univariate analysis			Multivariate analysis		
	OR	95 % CI	*P* value	OR	95 % CI	*P* value
Age at start of ICIs	1.00	[0.96, 1.05]	0.94			
Sex	0.77	[0.32, 1.96]	0.57			
BMI	1.01	[0.89, 1.13]	0.86			
Medical history						
Smoking status	0.78	[0.12, 2.93]	0.74			
Hypertension	0.47	[0.13, 1.33]	0.19			
Diabetes	1.00	[0.22, 3.23]	0.99			
Coronary heart disease	0.75	[0.04, 4.15]	0.79			
Metastatic site						
Liver	1.49	[0.57, 3.70]	0.40			
Lung	1.09	[0.40, 2.75]	0.85			
Bone	0.95	[0.26, 2.73]	0.92			
Brain	——	——	——			
Pre-existing treatment						
Chemotherapy	1.60	[0.63, 4.63]	0.35			
Radiotherapy	——	——	——			
Chemotherapy plus radiotherapy	0.87	[0.13, 3.31]	0.86			
None	0.61	[0.17, 1.74]	0.40			
Combination therapy						
Chemotherapy	0.78	[0.28, 1.94]	0.60			
Radiotherapy	——	——	——			
Targeted therapy	0.95	[0.38, 2.31]	0.91			
None	1.71	[0.53, 4.75]	0.33			
Time from ICIs to diagnosis	0.98	[0.85, 1.06]	0.68			
Co-existing other irAEs	7.44	[2.02, 25.95]	0.002	8.78	[2.03, 37.94]	0.004
ICIs						
Sintilimab	1.79	[0.73, 4.39]	0.20			
Camrelizumab	0.59	[0.13, 1.84]	0.41			
Tislelizumab	0.32	[0.02, 1.63]	0.27			
Toripalimab	1.66	[0.36, 5.57]	0.45			
Best observed response						
Complete response	1.68	[0.09, 11.19]	0.64			
Partial response	——	——	——			
Stable disease	0.99	[0.39, 2.59]	0.99			
Progressive disease	1.45	[0.54, 3.72]	0.45			
ORR	0.34	[0.02, 1.78]	0.31			
DCR	0.69	[0.27, 1.86]	0.45			
Blood routine test						
ΔWBC	1.15	[1.03, 1.30]	0.01	1.16	[1.01, 1.32]	0.04
ΔANC	1.21	[1.06, 1.38]	0.004	1.22	[1.05, 1.43]	0.01
ΔAEC	0.76	[0.14, 4.57]	0.76			
ΔAMC	——	——	——			
ΔALC	0.95	[0.45, 1.05]	0.82			
ΔPLT	1.00	[0.996, 1.005]	0.86			
ΔNLR	1.10	[1.03, 1.18]	0.01	1.11	[1.03, 1.21]	0.01
ΔLMR	0.94	[0.72, 1.02]	0.65			
ΔPLR	1.00	[0.999, 1.004]	0.23			
ΔSII	1.00	[1.0001, 1.0005]	0.003	1.00	[1.0001, 1.0006]	0.004
ΔSIRI	1.09	[1.01, 1.19]	0.03	1.10	[1.01, 1.21]	0.03
ΔLDH	1.00	[0.998, 1.002]	0.41			
ΔCRP	1.00	[0.99, 1.01]	0.76			
Cytokine						
ΔIL-1	1.07	[0.98, 1.21]	0.19			
ΔIL-2	0.95	[0.88, 1.04]	0.20			
ΔIL-6	1.00	[0.993, 1.008]	0.71			
ΔIL-8	1.00	[1.0001, 1.0032]	0.04			
ΔIL-10	1.01	[0.97, 1.07]	0.60			
ΔIL-17	1.01	[0.99, 1.04]	0.41			
ECHO						
ΔLVEF	0.87	[0.77, 0.96]	0.01	0.84	[0.74, 0.96]	0.01
ΔAOD	0.98	[0.75, 1.29]	0.91			
ΔLAD	0.98	[0.82, 1.17]	0.81			
ΔLVDD	1.08	[0.95, 1.26]	0.30			
ΔLVSD	1.10	[0.92, 1.30]	0.26			
ΔIVS	0.80	[0.36, 1.72]	0.57			
ΔLVPW	1.16	[0.47, 2.88]	0.74			
ΔS-wave peak	——	——	——			
Electrocardiography						
ΔHeart rate	1.00	[0.97, 1.04]	0.94			
ΔPR interval	1.01	[0.99, 1.02]	0.51			
ΔQRS duration	1.05	[1.00, 1.11]	0.04	1.06	[1.00, 1.12]	0.04
ΔQRS axis	1.00	[0.99, 1.01]	0.27			
ΔQT interval	1.00	[0.990, 1.007]	0.71			
ΔQTc	1.00	[1.00, 1.01]	0.12			
ΔRV5 + SV1	0.89	[0.42, 2.10]	0.77			

ICIs, immune checkpoint inhibitors; BMI, body mass index; irAEs, immune-related adverse events; CR, complete response; PR, partial response; SD, stable disease; PD, progressive disease; ORR, objective response rate; DCR, disease control rate; WBC, white blood cell count; ANC, absolute neutrophil count; AEC, absolute eosinophil count; AMC, absolute monocyte count; ALC, absolute lymphocyte count; PLT, platelet count; NLR, neutrophil-to-lymphocyte ratio; LMR, lymphocyte-to-monocyte ratio; PLR, platelet-to-lymphocyte ratio; SII, systemic immune-inflammation index; SIRI, systemic inflammation response index; LDH, lactate dehydrogenase; CRP, C-reactive protein; LVEF, Left Ventricular Ejection Fraction; AOD, Aortic Diameter; LAD, Left Atrial Diameter; LVDD, Left Ventricular Diastolic Diameter; LVSD, Left Ventricular Systolic Diameter; IVS, Interventricular Septum; LVPW, Left Ventricular Posterior Wall; OR, odds ratio; 95% CI, 95% confidence interval.

**Figure 3:**
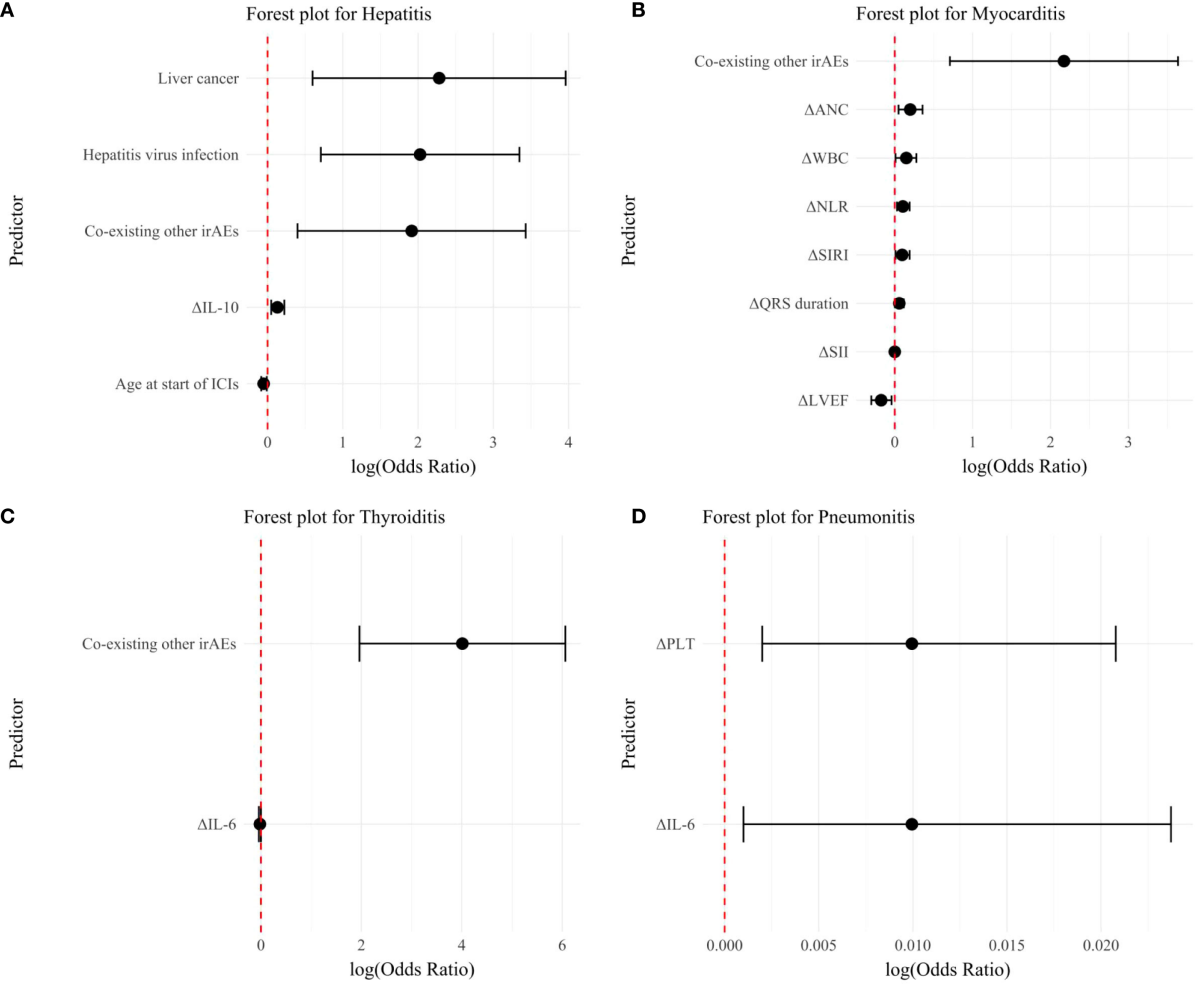
Forest plot showing independent predictors of organ-specific irAEs in patients treated predominantly with Chinese ICIs. **(A)** Immune-related hepatitis; **(B)** Immune-related myocarditis; **(C)** Immune-related thyroiditis; **(D)** Immune-related pneumonitis. irAEs, immune-related adverse events; ICIs, immune checkpoint inhibitors; ANC, absolute neutrophil count; WBC, white blood cell count; NLR, neutrophil-to-lymphocyte ratio; SIRI, systemic inflammation response index; SII, systemic immune-inflammation index; LVEF, Left Ventricular Ejection Fraction; PLT, platelet count.

Immune-related thyroiditis was significantly associated with the occurrence of two or more irAEs (OR: 55.24, 95% CI: 7.11-429.23, p < 0.001) and ΔIL-6 (OR: 0.98, 95% CI: 0.96-1.00, p = 0.04) (**Table 5**). Predictive factors for immune-related pneumonitis were identified in laboratory indices: ΔPLT (OR: 1.01, 95% CI: 1.002-1.021, p = 0.01) and ΔIL-6 (OR: 1.01, 95% CI: 1.001-1.024, p = 0.03) (**Table 6**). No significant predictive factors for immune-related hypophysitis were found (**Table 7**). A summary of these key factors significantly associated with each organ-specific irAE is presented visually (**Figure 3**).

**Table 5:** Univariate and multivariate analysis of factors for patients with immune-related thyroiditis.

Factors	Univariate analysis			Multivariate analysis		
	OR	95 % CI	*P* value	OR	95 % CI	*P* value
Age at start of ICIs	1.02	[0.97, 1.08]	0.54			
Sex	2.10	[0.63, 9.53]	0.27			
BMI	0.99	[0.85, 1.13]	0.91			
Medical history						
Smoking status	1.36	[0.20, 5.44]	0.70			
Hypertension	1.28	[0.38, 3.88]	0.67			
Diabetes	0.47	[0.03, 2.51]	0.48			
Coronary heart disease	1.27	[0.07, 7.32]	0.83			
Metastatic site						
Liver	1.42	[0.42, 4.30]	0.55			
Lung	1.32	[0.39, 3.98]	0.64			
Bone	0.70	[0.11, 2.71]	0.65			
Brain	——	——	——			
Pre-existing treatment						
Chemotherapy	2.20	[0.66, 9.96]	0.24			
Radiotherapy	——	——	——			
Chemotherapy plus radiotherapy	2.64	[0.56, 9.41]	0.16			
None	——	——	——			
Combination therapy						
Chemotherapy	0.67	[0.18, 2.07]	0.51			
Radiotherapy	——	——	——			
Targeted therapy	1.16	[0.38, 3.50]	0.79			
None	1.53	[0.33, 5.27]	0.53			
Time from ICIs to diagnosis	0.99	[0.85, 1.08]	0.92			
Co-existing other irAEs	14.68	[3.75, 56.12]	<0.001	55.24	[7.11, 429.23]	<0.001
ICIs						
Sintilimab	1.28	[0.41, 3.82]	0.66			
Camrelizumab	1.07	[0.23, 3.63]	0.92			
Tislelizumab	2.11	[0.45, 7.39]	0.28			
Toripalimab	0.74	[0.04, 4.08]	0.78			
Best observed response						
Complete response	2.78	[0.14, 19.20]	0.37			
Partial response	1.90	[0.28, 7.99]	0.43			
Stable disease	1.91	[0.60, 7.27]	0.30			
Progressive disease	0.16	[0.009, 0.840]	0.08			
ORR	2.28	[0.48, 8.22]	0.24			
DCR	6.27	[1.19, 115.53]	0.08			
Blood routine test						
ΔWBC	1.07	[0.92, 1.22]	0.37			
ΔANC	1.04	[0.88, 1.22]	0.60			
ΔAEC	0.55	[0.08, 4.75]	0.57			
ΔAMC	4.98	[1.05, 34.52]	0.10			
ΔALC	2.83	[1.10, 7.73]	0.04			
ΔPLT	1.00	[0.996, 1.007]	0.61			
ΔNLR	0.95	[0.82, 1.05]	0.45			
ΔLMR	1.09	[1.00, 1.34]	0.48			
ΔPLR	1.00	[0.994, 1.001]	0.30			
ΔSII	1.00	[0.9995, 1.0002]	0.58			
ΔSIRI	1.00	[0.97, 1.09]	0.89			
ΔLDH	1.00	[0.994, 1.001]	0.69			
ΔCRP	1.00	[0.98, 1.02]	0.87			
Cytokine						
ΔIL-1	0.95	[0.89, 1.00]	0.05			
ΔIL-2	0.99	[0.90, 1.14]	0.81			
ΔIL-6	0.99	[0.97, 1.00]	0.03	0.98	[0.96, 1.00]	0.04
ΔIL-8	1.00	[0.997, 1.000]	0.02			
ΔIL-10	1.00	[0.96, 1.06]	0.97			
ΔIL-17	0.98	[0.95, 1.00]	0.09			

ICIs, immune checkpoint inhibitors; BMI, body mass index; irAEs, immune-related adverse events; CR, complete response; PR, partial response; SD, stable disease; PD, progressive disease; ORR, objective response rate; DCR, disease control rate; WBC, white blood cell count; ANC, absolute neutrophil count; AEC, absolute eosinophil count; AMC, absolute monocyte count; ALC, absolute lymphocyte count; PLT, platelet count; NLR, neutrophil-to-lymphocyte ratio; LMR, lymphocyte-to-monocyte ratio; PLR, platelet-to-lymphocyte ratio; SII, systemic immune-inflammation index; SIRI, systemic inflammation response index; LDH, lactate dehydrogenase; CRP, C-reactive protein; OR, odds ratio; 95% CI, 95% confidence interval.

**Table 6:** Univariate and multivariate analysis of factors for patients with immune-related pneumonitis.

Factors	Univariate analysis			Multivariate analysis		
	OR	95 % CI	*P* value	OR	95 % CI	*P* value
Age at start of ICIs	1.05	[0.99, 1.13]	0.14			
Sex	5.85	[1.09, 108.53]	0.10			
BMI	1.04	[0.88, 1.19]	0.60			
Medical history						
Smoking status	1.84	[0.27, 7.76]	0.45			
Hypertension	1.97	[0.55, 6.79]	0.28			
Diabetes	4.07	[1.01, 14.55]	0.03			
Coronary heart disease	1.67	[0.09, 10.01]	0.64			
Lung cancer	2.81	[0.70, 9.85]	0.12			
Metastatic site						
Liver	0.54	[0.08, 2.17]	0.44			
Lung	1.35	[0.34, 4.65]	0.64			
Bone	1.66	[0.35, 6.05]	0.47			
Brain	4.11	[0.58, 18.85]	0.09			
Pre-existing treatment						
Chemotherapy	0.46	[0.13, 1.57]	0.21			
Radiotherapy	——	——	——			
Chemotherapy plus radiotherapy	3.69	[0.76, 14.08]	0.07			
None	1.09	[0.23, 3.92]	0.90			
Combination therapy						
Chemotherapy	0.63	[0.13, 2.24]	0.50			
Radiotherapy	——	——	——			
Targeted therapy	0.95	[0.27, 3.26]	0.94			
None	2.15	[0.45, 7.92]	0.28			
Time from ICIs to diagnosis	1.02	[0.86, 1.11]	0.74			
Co-existing other irAEs	4.11	[0.58, 18.85]	0.09			
ICIs						
Sintilimab	0.36	[0.05, 1.43]	0.19			
Camrelizumab	3.56	[0.98, 12.44]	0.04			
Tislelizumab	0.71	[0.04, 3.97]	0.75			
Toripalimab	——	——	——			
Best observed response						
Complete response	3.38	[0.17, 23.80]	0.29			
Partial response	0.99	[0.05, 5.68]	0.99			
Stable disease	0.98	[0.28, 3.50]	0.97			
Progressive disease	0.77	[0.16, 2.77]	0.71			
ORR	1.62	[0.24, 6.83]	0.55			
DCR	1.30	[0.36, 6.10]	0.71			
Blood routine test						
ΔWBC	0.87	[0.73, 1.04]	0.13			
ΔANC	0.85	[0.70, 1.03]	0.10			
ΔAEC	0.22	[0.03, 1.94]	0.13			
ΔAMC	5.43	[0.98, 45.43]	0.12			
ΔALC	0.91	[0.32, 1.06]	0.86			
ΔPLT	1.01	[1.00, 1.01]	0.05	1.01	[1.00, 1.02]	0.01
ΔNLR	0.99	[0.85, 1.07]	0.82			
ΔLMR	0.97	[0.67, 1.01]	0.85			
ΔPLR	1.00	[0.999, 1.005]	0.13			
ΔSII	1.00	[0.9998, 1.0004]	0.36			
ΔSIRI	1.05	[0.97, 1.14]	0.32			
ΔLDH	1.00	[0.98, 1.00]	0.82			
ΔCRP	1.01	[0.99, 1.02]	0.54			
Cytokine						
ΔIL-1	1.08	[0.97, 1.24]	0.22			
ΔIL-2	1.08	[0.92, 1.31]	0.45			
ΔIL-6	1.01	[1.00, 1.01]	0.08	1.01	[1.00, 1.02]	0.03
ΔIL-8	1.00	[0.999, 1.002]	0.61			
ΔIL-10	0.99	[0.95, 1.07]	0.86			
ΔIL-17	1.02	[0.99, 1.05]	0.34			

ICIs, immune checkpoint inhibitors; BMI, body mass index; irAEs, immune-related adverse events; CR, complete response; PR, partial response; SD, stable disease; PD, progressive disease; ORR, objective response rate; DCR, disease control rate; WBC, white blood cell count; ANC, absolute neutrophil count; AEC, absolute eosinophil count; AMC, absolute monocyte count; ALC, absolute lymphocyte count; PLT, platelet count; NLR, neutrophil-to-lymphocyte ratio; LMR, lymphocyte-to-monocyte ratio; PLR, platelet-to-lymphocyte ratio; SII, systemic immune-inflammation index; SIRI, systemic inflammation response index; LDH, lactate dehydrogenase; CRP, C-reactive protein; OR, odds ratio; 95% CI, 95% confidence interval.

**Table 7:** Univariate and multivariate analysis of factors for patients with immune-related hypophysitis.

Factors	Univariate analysis			Multivariate analysis		
	OR	95 % CI	*P* value	OR	95 % CI	*P* value
Age at start of ICIs	1.00	[0.94, 1.07]	0.88			
Sex	0.53	[0.12, 2.32]	0.39			
BMI	1.08	[0.91, 1.25]	0.31			
Medical history						
Smoking status	——	——	——			
Hypertension	0.31	[0.02, 1.81]	0.28			
Diabetes	0.90	[0.05, 5.37]	0.93			
Coronary heart disease	——	——	——			
Metastatic site						
Liver	0.82	[0.12, 3.70]	0.82			
Lung	2.41	[0.55, 10.52]	0.22			
Bone	0.60	[0.03, 3.52]	0.64			
Brain	6.27	[0.85, 31.56]	0.04			
Pre-existing treatment						
Chemotherapy	0.95	[0.23, 4.75]	0.95			
Radiotherapy	——	——	——			
Chemotherapy plus radiotherapy	3.14	[0.44, 14.77]	0.18			
None	0.40	[0.02, 2.33]	0.40			
Combination therapy						
Chemotherapy	——	——	——			
Radiotherapy	——	——	——			
Targeted therapy	8.57	[1.49, 161.80]	0.05			
None	0.77	[0.04, 4.54]	0.81			
Time from ICIs to diagnosis	1.06	[0.96, 1.16]	0.18			
Co-existing other irAEs	2.43	[0.12, 15.49]	0.43			
ICIs						
Sintilimab	0.55	[0.08, 2.44]	0.47			
Camrelizumab	1.32	[0.19, 5.96]	0.74			
Tislelizumab	2.54	[0.36, 11.78]	0.27			
Toripalimab	——	——	——			
Best observed response						
Complete response	5.77	[0.28, 44.21]	0.13			
Partial response	1.69	[0.09, 10.76]	0.64			
Stable disease	1.09	[0.23, 5.66]	0.91			
Progressive disease	0.34	[0.02, 2.03]	0.32			
ORR	2.99	[0.41, 14.88]	0.21			
DCR	2.97	[0.49, 56.87]	0.32			
Blood routine test						
ΔWBC	0.98	[0.79, 1.20]	0.86			
ΔANC	0.94	[0.74, 1.17]	0.58			
ΔAEC	3.41	[0.19, 37.05]	0.37			
ΔAMC	——	——	——			
ΔALC	——	——	——			
ΔPLT	1.00	[0.990, 1.006]	0.63			
ΔNLR	0.90	[0.74, 1.05]	0.29			
ΔLMR	——	——	——			
ΔPLR	1.00	[0.992, 1.001]	0.18			
ΔSII	1.00	[0.9993, 1.0002]	0.28			
ΔSIRI	0.99	[0.96, 1.09]	0.82			
ΔLDH	——	——	——			
ΔCRP	1.00	[0.98, 1.02]	0.76			
Cytokine						
ΔIL-1	1.03	[0.94, 1.21]	0.74			
ΔIL-2	1.15	[0.93, 1.44]	0.21			
ΔIL-6	1.00	[0.98, 1.01]	0.69			
ΔIL-8	1.00	[0.998, 1.002]	0.92			
ΔIL-10	1.01	[0.95, 1.10]	0.86			
ΔIL-17	1.01	[0.98, 1.05]	0.72			

ICIs, immune checkpoint inhibitors; BMI, body mass index; irAEs, immune-related adverse events; CR, complete response; PR, partial response; SD, stable disease; PD, progressive disease; ORR, objective response rate; DCR, disease control rate; WBC, white blood cell count; ANC, absolute neutrophil count; AEC, absolute eosinophil count; AMC, absolute monocyte count; ALC, absolute lymphocyte count; PLT, platelet count; NLR, neutrophil-to-lymphocyte ratio; LMR, lymphocyte-to-monocyte ratio; PLR, platelet-to-lymphocyte ratio; SII, systemic immune-inflammation index; SIRI, systemic inflammation response index; LDH, lactate dehydrogenase; CRP, C-reactive protein; OR, odds ratio; 95% CI, 95% confidence interval.

Among the five irAEs analyzed, no predictive role was found for the four Chinese ICIs or the best treatment outcomes for patients.

## Discussion

This study represents the first real-world analysis investigating predictors of irAEs in patients treated predominantly with Chinese ICIs. By focusing on a large cohort from routine clinical practice, the findings provide valuable evidence to guide risk stratification and intervention strategies in this specific treatment setting.

Comparison between patients with and without irAEs revealed that younger age was significantly associated with a higher risk of developing irAEs following ICI therapy. This may be attributed to the more robust immune regulatory function seen in younger individuals. However, the predictive role of age in irAEs remains controversial. Some studies suggest that age may lead to an increased incidence of irAEs or show no significant differences between age groups (24, 25). In fact, this study found that the predictive value of age for irAEs is primarily reflected in immune-related hepatitis, where younger patients are more prone to hepatic adverse reactions, while no significant statistical differences were observed for other types of irAEs. Additionally, this study discovered that patients with brain metastases had a higher incidence of irAEs, which may be related to the immune microenvironment of brain metastases. Tumor-associated myeloid cells are an important cellular component of the brain metastatic microenvironment, and they can be recruited from peripheral circulation and accumulate at the metastatic sites (26, 27). Following ICI therapy, these immune cells may overcome the suppressive effects of the brain metastasis microenvironment and over-activate the immune system, leading to the onset of irAEs.

This study identified various clinical and laboratory factors significantly associated with PFS in ICI-treated patients. Specifically, higher age at ICI initiation and later onset of irAEs were associated with longer PFS, whereas liver metastasis predicted shorter PFS, and the absence of combination therapy was associated with longer PFS. Additionally, peripheral blood markers such as ΔNLR, ΔPLR, ΔSII, ΔSIRI, and ΔIL-8 were found to be significantly associated with poorer survival outcomes. These laboratory markers reflect the degree of systemic inflammation, and enhanced inflammatory responses may promote tumor angiogenesis and metastasis, which could explain the poorer prognosis (28, 29). Although several studies have suggested that irAEs are associated with extended PFS and overall survival (OS) in ICI-treated patients, the results of this study did not confirm that irAEs are significant predictors of therapeutic efficacy (30, 31). One study indicated that in patients with head and neck squamous cell carcinoma (HNSCC) receiving ICI therapy, the association between irAEs and treatment efficacy may be overestimated. After controlling for immortal time bias, it was found that irAEs were an independent predictor of OS but not PFS (32). The present study also applied time-dependent Cox models to account for immortal time bias and similarly found no significant association between irAE occurrence and PFS. Moreover, a meta-analysis concluded that when irAEs affect the skin, endocrine organs, or gastrointestinal tract, patients tend to experience significant survival benefits, whereas irAEs involving the liver and lungs do not exhibit similar advantages (33). The results are consistent with the meta-analysis, indicating no survival benefit from irAEs involving the liver and lungs.

This study analyzed the predictive factors for five major irAEs, and the results indicated that, except for pituitary inflammation, potential independent risk factors were found for all other irAEs. For patients who developed hepatitis, younger age at the start of ICI treatment, hepatitis B virus infection, hepatic malignancy, the presence of multiple irAEs, and ΔIL-10 were identified as risk factors. The independent predictors for myocarditis included the presence of multiple irAEs, ΔWBC, ΔANC, ΔNLR, ΔSII, ΔSIRI, ΔLVEF, and ΔQRS duration. The occurrence of thyroiditis was significantly associated with the presence of multiple irAEs and lower ΔIL-6, while the development of pneumonitis was linked to higher ΔPLT and ΔIL-6. Some of the findings in this study are consistent with previous research, though some independent predictive factors have rarely been reported before (34–39). Notably, several peripheral inflammatory markers, including ΔNLR, ΔSII, and ΔSIRI, were identified as predictors for both shorter PFS and the occurrence of myocarditis, whereas other predictors for PFS and major irAEs did not overlap.

Many cytokines have been implicated in the occurrence of irAEs, especially some pro-inflammatory cytokines (including IL-1, IL-6, IL-8, IL-17), where elevated baseline serum levels may serve as predictive biomarkers for irAEs (40–42). The results of this study suggest that cytokines have organ-specific predictive value for the occurrence of irAEs, with different types of irAEs corresponding to distinct changes in cytokine levels. This finding aligns with the views of Wang et al., although their conclusion that elevated ΔIL-6 can predict thyroiditis differs from the findings in this study (43). Existing research suggests that the onset of irAEs may result from multiple mechanisms, including autoreactive T cells, autoantibodies, and inflammatory cytokines, all of which are involved in immune-related pathways (10). For instance, T cell activation can induce the release of inflammatory cytokines, thus promoting the occurrence of irAEs. However, in this study, a decrease in ΔIL-6 was found to be an independent risk factor for thyroiditis, which does not align with the aforementioned mechanism. Some studies have reported that thyroiditis is characterized by CD8+ T cell infiltration and follicular structure destruction, suggesting that T cell-mediated cytotoxicity is a key mechanism (44). The pathogenesis of thyroiditis is influenced by the type of ICI used, and there are significant differences in cytokine profiles induced by different immunotherapy regimens (45). Most of the patients included in this study received Chinese ICIs, which may explain the contradictory results. More cellular and molecular studies are needed to clarify the role of cytokines in thyroiditis.

Given the convenience of peripheral blood cell tests and inflammation markers in clinical settings, numerous studies have reported their correlation with the occurrence of irAEs (37–39). In this retrospective study, the predictive value of peripheral blood tests was primarily observed in myocarditis. The results showed that the elevation of various peripheral blood markers, including ΔWBC, ΔANC, ΔNLR, ΔSII, and ΔSIRI, was an independent risk factor for myocarditis. This is a surprising finding, as previous studies on the correlation between myocarditis and peripheral blood markers have mostly reported only one or two markers with significant statistical differences (37, 38). This study found that multiple peripheral blood markers were independently associated with myocarditis, and the results are largely consistent with existing literature, suggesting that peripheral blood markers may serve as potential biomarkers for predicting myocarditis. The European Society of Cardiology (ESC) currently recommends troponin levels and electrocardiogram (ECG) tests for suspected myocarditis patients. Some studies have also found that combining troponin with ECG helps in the early screening of myocarditis (46, 47). However, predictive ECG markers for sinus rhythm myocarditis patients are still lacking. This study is the first to report that prolonged QRS duration can be an independent risk factor for myocarditis. Based on the above findings, this study suggests that peripheral blood markers, when combined with troponin and ECG, could improve the sensitivity of early myocarditis screening.

Research on multi-system irAEs is mostly case reports, and few cohort studies have found that patients with multi-system irAEs tend to have longer PFS and OS compared to those with single irAEs or no irAEs (48–50). In this retrospective study, the most common multi-system irAEs were hepatitis and myocarditis. This pattern differs from that reported by Shankar et al., who observed pneumonitis with thyroiditis, and by Zhang et al., who observed thyroiditis with dermatitis (48, 49). Tumor type and the ICI regimen used may be key factors influencing the differences in multi-system irAEs patterns (50). However, there is still a lack of studies on whether multi-system irAEs can predict the risk of specific irAEs. This study, through multivariate regression analysis, observed that patients with multi-system irAEs were more likely to develop hepatitis, myocarditis, and thyroiditis, suggesting their potential predictive value for specific irAE occurrences.

This study also evaluated the predictive effects of the four Chinese ICIs on organ-specific irAEs but found no significant associations. The absence of organ-specific toxicity signals is reassuring. However, the analysis may be underpowered due to the small number of cases in certain irAE subgroups. For example, the small sample size for hypophysitis limits definitive conclusions. Previous studies have suggested that the incidence of irAEs is significantly higher with sintilimab and camrelizumab compared to toripalimab and pembrolizumab; however, their specific predictive value has yet to be investigated (51). To our knowledge, this is the first retrospective study to evaluate the predictive value of Chinese ICIs for irAEs in a real-world patient cohort receiving ICI therapy. These findings indicate that the development of irAEs associated with Chinese ICIs may be influenced by multiple factors, rather than being determined solely by the pharmacological properties of the drugs. For clinicians, it is important to comprehensively consider the efficacy and irAE risk profiles of different Chinese ICIs when making treatment decisions, in order to optimize adverse event management.

However, this study has several limitations. First, as a single-center retrospective study, it is subject to potential information and selection bias. Second, the overall sample size was relatively limited, and the number of cases with certain types of irAEs was small, preventing multivariable regression analysis in some subgroups and limiting the generalizability of the conclusions. In addition, although irAEs vary in clinical presentation and severity, this study did not stratify irAEs according to CTCAE grading, which may have masked specific predictive factors for severe irAEs. Finally, despite identifying several potential predictive factors and mechanisms, this study lacks *in vitro* or animal experiments for further validation.

## Conclusion

This study examined irAEs and their predictors in patients predominantly treated with Chinese ICIs. Younger age and brain metastases were associated with a high incidence of irAEs. Organ-specific irAEs showed distinct predictors: hepatitis with age, HBV, liver cancer, multisystem irAEs, and ΔIL-10; myocarditis with multisystem irAEs, ΔWBC, ΔANC, ΔNLR, ΔSII, ΔSIRI, ΔLVEF and ΔQRS duration markers; thyroiditis with low ΔIL-6; pneumonitis with high ΔPLT and ΔIL-6. Inflammatory markers correlated with poorer PFS, whereas the type of irAEs was not associated with PFS. The type of Chinese ICIs was not an independent predictor of organ-specific irAEs.

## Data Availability

The datasets presented in this article are not readily available because The dataset contains anonymized patient information and is available upon reasonable request, subject to approval by the ethics committee to ensure compliance with privacy regulations. Requests to access the datasets should be directed to Zhuqing Liu, zhuqingliu2013@163.com.
