# Supplementary material for: Predictive factors and outcomes of immune-related adverse events in Chinese patients treated with immune checkpoint inhibitors: a real-world retrospective study
Source: Front Immunol. 2025 Sep 23;16:1655724. doi: 10.3389/fimmu.2025.1655724 (PMC12500697; doi:10.3389/fimmu.2025.1655724)
Supplement: Supplementary file 1 [file Table1.docx]

**Supplementary materials**

**Supplementary Table 1. Time-Dependent Univariate and Multivariate Analysis of irAEs and Progression-Free Survival**

| **Factors** | **Univariate analysis** | | |  | **Multivariate analysis** | | |
| --- | --- | --- | --- | --- | --- | --- | --- |
|  | **HR** | **95 % CI** | *P* **value** |  | **HR** | **95 % CI** | *P* **value** |
| **irAEs** | 1.21 | [0.80, 1.82] | 0.36 |  | 1.04 | [0.67, 1.61] | 0.87 |
| **Hepatitis** | 1.98 | [1.13, 3.47] | 0.02 |  | 1.24 | [0.67, 2.27] | 0.49 |
| **Myocarditis** | 1.27 | [0.52, 3.12] | 0.60 |  | 1.47 | [0.58, 3.74] | 0.42 |
| **Thyroiditis** | 0.56 | [0.21, 1.54] | 0.26 |  | 0.66 | [0.23, 1.89] | 0.44 |
| **Pneumonitis** | 0.70 | [0.22, 2.23] | 0.55 |  | 0.97 | [0.26, 3.64] | 0.96 |
| **Hypophysitis** | —— | —— | —— |  | —— | —— | —— |

**Supplementary Table 1.** Time-Dependent Univariate and Multivariate Analysis of irAEs and Progression-Free Survival. PFS, progression-free survival; HR, hazard ratio; 95% CI, 95% confidence interval.
